# Like or Dislike? Impact of Facebook on Ewing Sarcoma Treatment

**DOI:** 10.2196/cancer.5367

**Published:** 2016-08-25

**Authors:** Paul Ruckenstuhl, Michael Schippinger, Paul Liebmann, Andreas Leithner, Gerwin Bernhardt

**Affiliations:** ^1^ Department of Orthopedic Surgery Medical University Graz Graz Austria; ^2^ Department of General Surgery Evangelisches Krankenhaus Vienna Austria

**Keywords:** social media, Facebook, Ewing sarcoma, social media networking

## Abstract

**Background:**

An increasing number of patients are raising their voices in online forums to exchange health-related information. Facebook is the leading social media platform with more than 1 billion international daily users recorded in the summer of 2015. Facebook has a dynamic audience and is utilized in a number of ways, discussing medical issues being one of them. Ewing sarcoma mainly affects teenagers and young adults. Additionally, many individuals within this age group are regular users of Facebook. However, little is known about the impact of this modern way of communication via Web-based platforms on patients with Ewing sarcoma and their social environment.

**Objective:**

The aim of this study was to analyze and compare Ewing sarcoma patients’ and relatives’ behavior on Facebook to draw conclusions regarding the impact of Facebook on Ewing sarcoma treatment.

**Methods:**

We examined a Facebook group named “Ewing Sarcoma Awareness” that is used to exchange information for both patients and relatives regarding Ewing sarcoma. A self-designed questionnaire was used to compare patients’ and relatives’ answers. Additionally, we analyzed all processes (posts, likes, threads, links) in the group for 6 consecutive months. A total of 65 members of the Facebook group (26 patients, 39 relatives) out of 2227 international group members participated in our study.

**Results:**

More than 70% (46/65) of all participants reported that they use the group Ewing Sarcoma Awareness as a source of information about Ewing sarcoma. Of the participants, 89% (58/65) agreed on our scale from a little to a lot that being in contact with other affected people through the group makes it easier to handle the diagnosis. In this study, 20% (13/65) of all participants reported that the group affected their choice of treatment and 15% (10/65) of participants were influenced in the selection of their specialist. Regarding the recommendation of the Facebook group toward other people, significant differences (*P*=.003) were found comparing patients’ and relatives’ results. During the last 6 months most activities in the group concerned sharing destiny and handling the diagnosis.

**Conclusions:**

The Facebook group Ewing Sarcoma Awareness has a relevant impact on group members regarding their choice of treatment. Moreover, participants turn toward the group to receive mental and emotional support in everyday life. Statements made within the group are in part questionable from a medical point of view and the impact made by these statements on patients’ care requires further evaluation.

## Introduction

Web-based communication is a convenient method of exchanging information regarding health and well-being and is thus increasingly growing in popularity and commonly used [1,2]. Because it is ubiquitous and easy to use, the Web has become the number one source for patients to gather information on health-related issues [2-5].

The term “Web 2.0” describes an interactive way of using the Web by exchanging information via blogs, platforms, podcasts, wikis, and online forums. These tools offer possibilities to simplify Web-based communication between Web users. In this way, the Web is not only a platform to acquire information from websites passively, but also a viable asset to create and share knowledge [6]. Furthermore, Web 2.0 enables users to collaborate by distributing information [7]. This rapidly growing way of using the Web has brought Web-based communication to a new level on social media platforms [2,8,9].

More precisely, the Web enables both experts and laymen to discuss and promote health-related information. Patients are increasingly using social media sites to share sorrow, to exchange information about handling their daily routine, and to discuss treatment options using evidence-based standard therapeutic regimens for various kinds of diseases. This development enables the creation of an active, self-managing, and responsible “expert patient” [10,11]. However, it seems reasonable then that patients might run the risk of receiving pseudoscientific and incorrect information [2].

The leading social media platform presenting medical issues is Facebook (FB). With a record of more than 1 billion active users per day in August 2015, the website FB is besides “Google” the second most viewed site in the world [12]. Facebook is the most frequently used Web-based communication platform [2,13-15]. In 2008, a study reported that 45% of medical trainees, 64% of medical students, and 13% of medical residents have FB accounts [16]. Among US adults, 61% search for health information on the Web, of whom 39% use social media such as FB for health-related information [17]. Considering the growth of FB during the last years these numbers can, therefore, be estimated even higher [15]. Because of its enormous accessibility especially for rare diseases like Ewing sarcoma, FB is a ubiquitous and easy way to connect people with others affected [15,18-20].

The peak incidence of Ewing sarcoma is between 10 and 20 years of age and coincides with the main age group of FB users [21-24]. A study by Duggan and Brenner [25] reported that 86% of all Web users aged between 18 and 29 years use FB, thus making FB an ideal platform for patients with Ewing sarcoma to connect with each other.

Ewing sarcoma is the second most common bone sarcoma after osteosarcoma with an incidence of 1 case per 1 million people [21]. The treatment of choice is neoadjuvant chemotherapy followed by a wide resection of the tumor and adjuvant chemotherapy [21-23]. Survival of patients following this therapy regimen has increased, and two-thirds of patients are cured of their disease [21]. With a 5-year survival rate of 78% for children younger than 15 years and 60% for adolescents aged 15 to 19 years, Ewing sarcoma remains a severe diagnosis [21].

The diagnosis Ewing sarcoma poses an enormous challenge for young patients, their families, and their social environment. Because of its severity, Ewing sarcoma requires a treatment concept including also psychological aspects. It is well reported that cancer patients profit from peer-to-peer communication [15,26]. These days the Web provides various possibilities to get in contact with fellow sufferers, especially for rare diagnoses like Ewing sarcoma.

It is well known that the Web, particularly social media platforms, offers new dimensions to communication related to medical topics [2,27]. We believe that this way of communication has a relevant influence on the treatment regimen, the choice of consultant, particularly the choice of hospital, and dealing with the disease in general. Furthermore, this hypothesis might be underestimated in traditional treatment concepts.

However, little is known about patients’ and relatives’ behavior on social media platforms regarding Ewing sarcoma. The aim of this study was to examine the influence of interactive Web-based exchanges of information on the FB group “Ewing Sarcoma Awareness” (ESA) for patients with Ewing sarcoma and their relatives.

## Methods

### Facebook as a Search Engine

The most common way for a large number of people to communicate on FB is through “FB groups.” Facebook groups can be created by all FB users to communicate with a defined group of members about certain topics. To become a member of the group, one can either request to be a member or get an invitation from the group administrator.

In March 2014, we carried out a search for the term “Ewing sarcoma” using the FB search engine. The FB group used in this study is called Ewing Sarcoma Awareness. Ewing Sarcoma Awareness was by far the largest group we found for open Web-based communication for people affected by Ewing sarcoma. Ewing Sarcoma Awareness is defined as a public group and is available to all people with FB accounts. The group has two administrators who are able to control processes in the group. Facebook users are free to follow the group and to view all activity happening on the home page without being a group member. To become a member of the group a request has to be sent to the group administrators who grant admission to the group. The administrators are also able to remove group members or contributions posted to the page.

**Figure 1:**
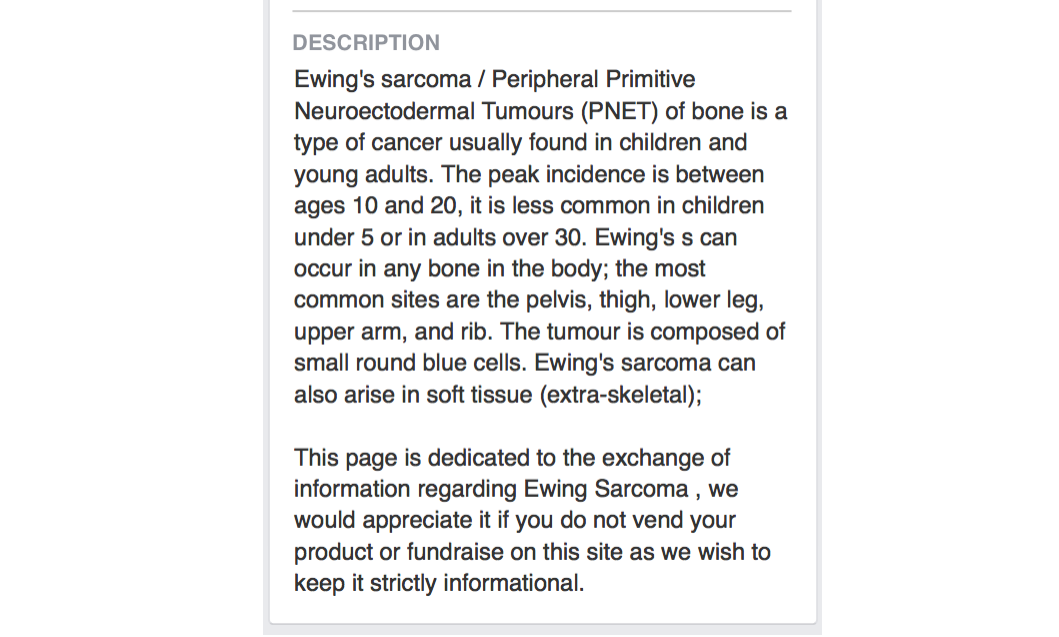
Screenshot of the description of the Ewing Sarcoma Awareness group on Facebook.

The ESA group’s main purpose, as declared in its description, is to facilitate the exchange of information regarding Ewing sarcoma for patients and other people affected by the disease (Figure 1). The exchange of information in the ESA group is mainly based on the home page of the group, where only members are able to post contributions. The types of contributions on the home page range from personal opinions, statements, pictures, and videos to recommendations regarding treatment options, clinical trials, research results, hospitals, doctors, and much more. Members are able to comment, like, or add something to these posts creating lively discussions. To deepen the exchange of information, members are able to use FB chat to communicate via private messages that cannot be seen by other members.

At the time of our investigation, the group consisted of 2227 international members. Most group members indicated that they live in the United States or Canada, although group members were from countries in all continents of the world. The group administrators and creators did not appear to be medical professionals or associated with health care institutions or organizations [28].

**Figure 2:**
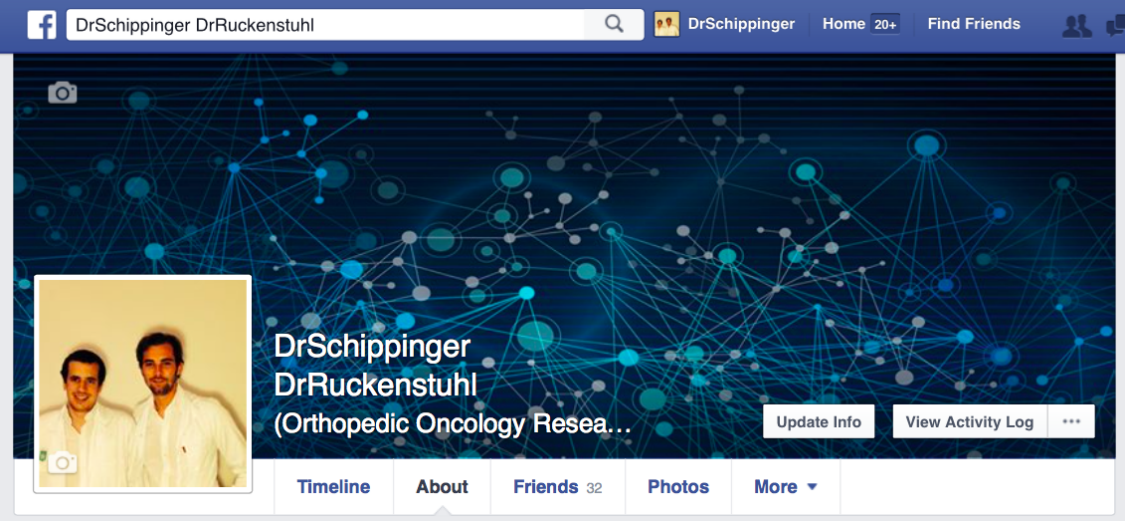
Screenshot of the home page of our research Facebook profile.

To get in contact with the group we created a FB profile that introduced ourselves to the social media community (Figure 2). Our FB profile became a group member of ESA group after confirmation of request by one of the group administrators.

### Questionnaire

We created a Web-based questionnaire using “SurveyMonkey” to analyze the group members’ behavior regarding their use of the Web and FB for people affected by Ewing sarcoma. SurveyMonkey.com was founded in 1999, and with more than 20 million users worldwide it is one of the leading platforms for Web-based surveys [29]. It offers tools to create and analyze Web-based surveys [29].

The self-designed questionnaire was developed on the basis of the “Checklist for Reporting Results of Internet E-Surveys” [30]. The survey was designed as an open survey for all ESA group members, consisted of 18 multiple-choice questions (Q), and was divided in 4 categories. The first category (Q1-Q4) dealt with the user’s behavior in the FB group. Categories 2 (Q5-Q8) and 3 (Q9-Q13) were composed of questions concerning the reliability and quality of information received in the group. Additionally, questions asking about the effects that ESA group has had on the user’s decision-making processes were included. The last category (Q14-Q18) consisted of questions about the user’s general activity on the Web regarding medical and health-related issues. Users answered by rating each statement on a scale ranging from 1 to 4 (1=disagree a lot, 2=disagree a little, 3=agree a little, 4=agree a lot).

Accompanying this survey was background information of participants regarding sex, age, and whether participants were patients or their relatives or friends. Answering all questions took approximately 4 to 7 minutes.

**Figure 3:**
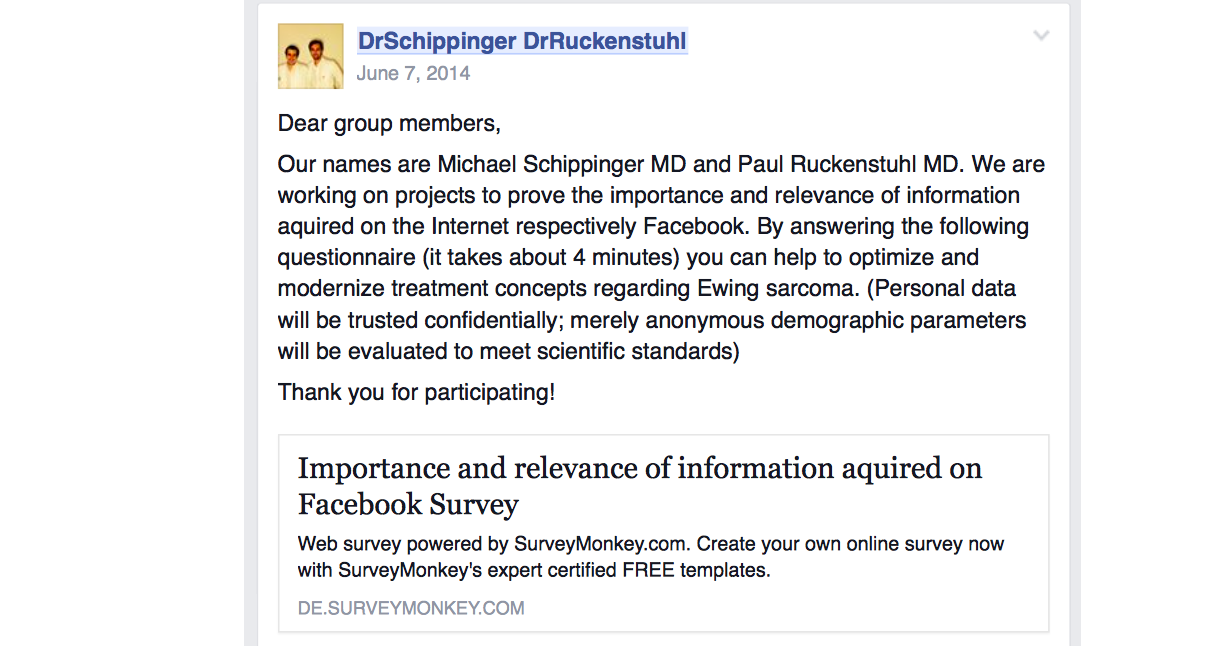
Screenshot of our survey request on the Ewing Sarcoma Awareness group’s title page.

We posted a link concerning our Web-based survey on the ESA group’s home page on FB (Figure 3). Additionally, we explained the study’s purpose. On August 23, we posted again in the group to re-invite all group members to participate and maximize study sample before we closed the link by the end of August 2014. To clarify obscurities or other kinds of questions we corresponded with group members via private FB messages.

All responses were automatically recorded via the Web-based survey platform [29].

### “Ewing Sarcoma Awareness” Group Analysis

Besides the questionnaire, we analyzed all processes happening in the ESA group over a 6-month period (October 2013 to March 2014). The analysis referred to activities in the group, more specifically to threads and contributions that were posted on the group’s home page. We started by analyzing the content of each wall post to develop a classification scheme that could be applied to the complete observational time period.

All posts were divided into 2 main groups: informative and emotional contributions. We then further subdivided the 2 main groups each into 3 branches (Table 1).

**Table 1:** Classification of posts on the Ewing Sarcoma Awareness group’s home page.

Categories and subcategories		Description

**Emotional**		
	Sharing fate/getting support	Group members are reporting about their case/destiny or occurring problems
	Bereavement/recurrence	Group members are reporting the loss of a relative or child, or the recurrence of the disease
	Complete remission/ no evidence of disease	Group members are reporting about successful treatments
**Informative**		
	Information	Group members are asking for helpful advice to handle the disease and the occurring side effects under the therapy
	Clinical trials	Group members are asking for new trials or are sharing information about new studies
	Recommendations	Group members are reporting about their experiences and satisfaction/dissatisfaction, or are asking for specialists/hospitals in their area

After classifying the intentions of all posts, we evaluated the number of postings in each subgroup. Contributions deleted by the administrator or those without any classifiable content were excluded from our analysis. Three of the authors coded the data independently according to Table 1; in case of a disagreement, the coding was discussed in the group.

### Statistics

Statistical analysis was performed comparing patients’ and relatives’ values for each of the 18 questions using *t* test. Parametrically distributed data are described as the mean and the standard deviation (SD). All tests were 2-sided with a significance level of *P*<.05. Pearson and Spearman correlation were performed where appropriate.

For statistical calculations SPSS version 22 (IBM Statistics, SPSS Software, IBM Vienna, Austria) was used. Data of all participants were anonymized. As all the information is publicly available, no review by an institutional research ethics board was needed.

## Results

### Survey Results

The study group of our survey consisted of 65 participants: 26 patients and 39 relatives or friends of patients. Incomplete surveys (n=26) were excluded. Of the participants, 11 were male (mean 37.4, SD 14.4 years) and 54 were female (mean 39.8, SD 10.4 years). Average age of the patients was mean 32.9 (SD 8.4) years and that of the relatives was mean 43.6 (SD 10.1) years (Table 2).

**Table 2:** Age characteristics of study participants (N=65).

Age, years	Patients	Relatives
20-25	4	1
26-30	8	4
31-35	4	6
36-40	4	5
>40	6	24
Total, n (%)	26 (40)	39 (60)

The highest values for patients and relatives with a mean score of 3.01 (SD 0.87) were found in the first category of the questionnaire that focused on the users’ behavior. The second category that concerns the influence of the ESA group on participants’ therapeutic schedule yielded the lowest results for patients and relatives with a mean score of 1.91 (SD 0.91).

The maximum mean score per question for patients was found in question 15 with 3.65 (SD 0.69), followed by question 4 (mean 3.54, SD 0.58) and question 9 (mean 3.50, SD 0.76). Lowest agreements for patients were found in question 7 with a mean score of 1.46 (SD 0.86) followed by questions 6 (mean 1.54, SD 0.81) and 5 (mean 1.69, SD 0.97).

In addition, we compared the given answers from patients and relatives (Table 3). There was a statistically significant difference between patients and relatives concerning question 4 (mean 3.5, SD 0.6 vs mean 3.0, SD 0.9; *P*=.003). All the given answers of the survey significantly correlated with each other as well as within the patients' and relatives' groups (*P*<.001).

**Table 3:** Survey results of patients and relatives.

No.	Question	Patients		Relatives		*P*
		Mean	SD	Mean	SD	
1.	I frequently (4=daily, 3=weekly, 2=monthly, 1=less) visit the Facebook group “Ewing sarcoma awareness” to be in contact with other affected people.	2.85	1.12	2.87	0.92	.92
2.	I post, comment, or like activities in the group or contact other group members via private messages.	2.92	0.89	2.79	0.83	.56
3.	I use the Facebook group “Ewing sarcoma awareness” as a source of information about Ewing sarcoma.	3.04	0.96	3.10	0.79	.78
4.	I recommend the Facebook group “Ewing sarcoma awareness” in other social networks or to other affected people.	3.54	0.58	2.97	0.90	.003


5.	The information I received in the Facebook group “Ewing sarcoma awareness” affected the choice of treatment.	1.69	0.97	1.69	0.80	.99
6.	The information I received in the Facebook group “Ewing sarcoma awareness” affected my choice of consultant.	1.54	0.81	1.67	0.77	.53
7.	The reliability of my consultant decreased because of information I received in the Facebook group “Ewing sarcoma awareness.”	1.46	0.86	1.56	0.79	.63
8.	I never had the experience that wrong information in the group “Ewing sarcoma awareness” led to a negative dealing with the disease.	2.96	1.11	2.74	1.23	.46


9.	Being in contact with other affected people via the Facebook group “Ewing sarcoma awareness” makes it easier to handle the diagnosis of Ewing sarcoma.	3.50	0.76	3.49	0.72	.95
10.	I received useful information in the Facebook group “Ewing sarcoma awareness,” which improved my everyday life in dealing with the disease.	3.23	0.86	3.03	0.84	.35
11.	I trust the Facebook group “Ewing sarcoma awareness” to receive correct information about Ewing Sarcoma.	2.96	0.87	3.03	0.78	.76
12.	The Facebook group “Ewing sarcoma awareness” is an important support for me to handle the disease.	3.27	0.87	3.18	0.79	.68
13.	I received information about new clinical trials as well as specialists through the Facebook group “Ewing sarcoma awareness.”	2.38	1.16	2.28	0.94	.71


14.	I take part in other Ewing sarcoma groups or forums on the Internet, including other social media platforms.	3.23	0.99	2.85	1.11	.16
15.	The Internet is an important tool for me to look for information about Ewing sarcoma.	3.65	0.69	3.49	0.85	.39
16.	I trust online platforms like Wikipedia, Twitter, YouTube, and Facebook to receive correct information about Ewing sarcoma.	2.42	0.95	2.36	0.99	.79
17.	I generally look for information about diseases on the Internet prior to consultation.	3.00	1.01	3.15	0.87	.53
18.	The Internet is an important source for me to look for health-related information.	3.27	0.83	3.28	0.79	.95

### Processes in the Group

A total of 220 posts on the ESA group’s home page and 445 comments were included and categorized accordingly.

**Figure 4:**
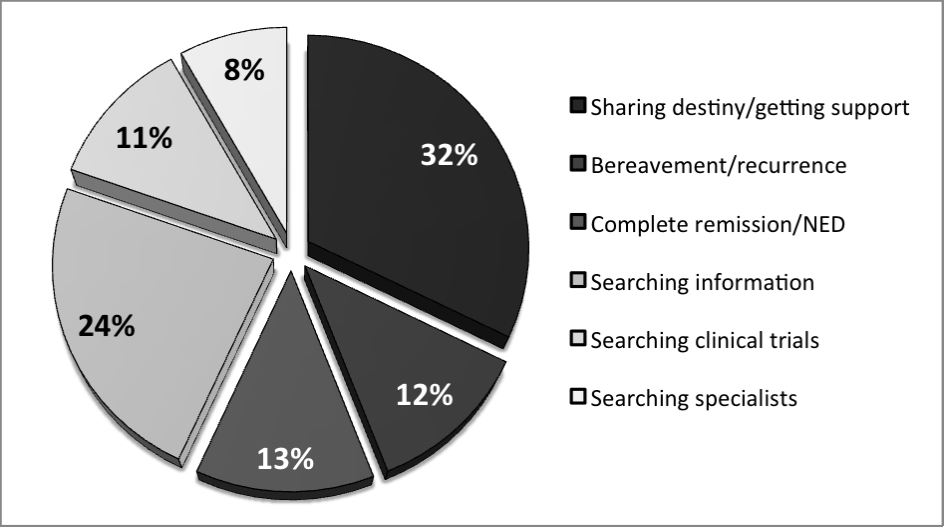
Results of posts on the Ewing Sarcoma Awareness group’s home page.

We detected a total number of 453 home page posts and 917 comments from 183 different group members. Because of lack of relevance or information, 233 posts and 472 comments were excluded from our study. These were reaction comments to previous posts or posts about everyday topics, not specific to Ewing sarcoma. The posts’ contents addressed subjects such as dietary supplements under chemotherapy, correct behavior as a family member, introduction to the group, and others. Overall, 125/220 (56.8%) posts in the ESA group were categorized as emotional posts and 95/220 (43.2%) as informative contributions. As shown in Figure 4, most topics discussed were about sharing destiny (71/220 posts, 32.3%). Other posts that were assigned to the subgroup of emotional contributions were about the disease’s relapse, particularly, bemoaning the loss of a relative (26/220 posts, 11.8%) and reports of complete remission (28/220 posts, 12.7%). The most informative contributions were about searching for Ewing sarcoma–related information (52/220 posts, 23.6%). Group members asked for recommended specialists, hospitals, and reports of experience (25/220 posts, 11.4%). Members also discussed posted hyperlinks with information about new clinical trials (18/220 posts, 8.2%).

Moreover, 15 articles about research results, fund raising, or donations were discussed in the forum. Some of these articles were deleted during the period of our observation by the group administrator.

## Discussion

### Principal Findings

With respect to our principal findings, the Facebook group Ewing Sarcoma Awareness has a relevant impact on group members regarding their choice of treatment. Moreover, participants turn toward the group to receive mental and emotional support in everyday life. Reflecting on our results, we believe that a better understanding of this growing interest in peer-to-peer communication for patients may lead to an optimization of a patient-related therapeutic regimen.

The Web has rapidly grown to be one of the leading sources of medical information. It is well known that the Web, in particular social media communication, brings a new dimension to medical subjects followed by possibly improving health outcomes [2]. The idea of this study was to analyze users’ behavior regarding Ewing sarcoma on the social media site FB.

Several studies have described users’ behavior on FB for health-related issues [15,20,26,31-34]. These studies mainly compared FB groups and/or analyzed processes happening in the group regarding a wide range of different diseases [35]. To the best of our knowledge, there is no study to date that used a comparable approach to interact with FB group members as we did. Therefore, a comparison with other reports was not possible. Yet, we believe that the direct interaction via Web-based surveys with members of FB groups adds a new methodical modality in medical Internet research.

Facebook is a ubiquitous social media platform including health-related issues. Bender et al [18] examined FB groups related to breast cancer. The findings from their study confirmed our presumption that FB is a popular tool for millions of users to seek support via social media platforms. Abramson et al reported about a breast cancer awareness page on Facebook that underlines the increasing use of Facebook pages to discuss severe medical conditions via social media platforms [20]. The visibility of user profiles and personal networks in open FB groups like the ESA group reduces the anonymity but attracts a much wider audience. These key elements of social network sites make public groups ideally suited for fundraising and awareness-raising purposes [18]. Compared with the findings of Bender et al [18], fundraising was of lesser importance in the ESA group. Moreover, marketing and promotion as found by Hale and colleagues [35] played a minor role in our study. General information about the disease, sharing faith, personal support, and assistance in how to handle their daily routine were more important factors reflected in our findings. This might be associated with the rare prevalence of Ewing sarcoma. Different studies reported that FB members use the social media site as a source of information for health-related issues [36,37]. Other studies concluded that FB plays a less important role and has little relevance regarding health-related Web-based information [2,38].

The group ESA was by far the biggest platform (n=2227) we found on FB to exchange general information about Ewing sarcoma. Most other contributions about Ewing sarcoma on FB are blogs about fates of individuals and nonprofit institutions created for fundraising.

It is notable that more than 80% (54/65) of all participants of our Web-based survey were female. Pennbrige et al [39] support this observation and found that 60% of US Internet users using the Web to gather health-related information were women. Most likely, due to caretaking roles and behavior, women appear to visit health-related webpages more frequently [39]. This is consistent with several other studies that reported females regularly visiting social networking sites for the acquisition of health-related information [40-42].

More than half of all participants were aged 36 years or older and 30/65 participants (45%) were older than 40 years. This explains why mainly parents of patients participated in the relative sample group. This is consistent with the incidence peak of the disease in the teenage years. However, the age of participants of our study differed from the age of participants of most other FB research studies [43,28]. No teenagers participated in our questionnaire although the prevalence of Ewing sarcoma as well as the core age group of FB users would correspond to this age. A possible reason for this could be that the appearance of our FB profile did not attract enough attention for the young group members to participate.

According to the findings of Davison et al [44], social media platforms are less attractive for medical conditions considered to be embarrassing and socially stigmatizing. This also might discourage the adolescent age group to participate in our Web-based survey. Unlike in our study, the average age of participants of other scientific works ranged from 11 to 34 years [41,42,45,46].

The Web in general has become the number one source of medical information for many patients [2]. Referring to the results of our survey, 84% of all participants agreed a little or a lot that the Web is an important source for health-related information (Q18). Moreover, 77% (50/65 participants) reported that they use the Web to look up medical conditions and symptoms before medical consultations (Q17). These findings are in accordance with several studies about public and patients’ behavior on the Web regarding medical issues [2,11,20,24,47,48].

Reliability and quality of health-related information found on the Web is considered generally questionable. According to our survey, 47% (30/65) of all participants agreed a little or a lot that they trust the information available on Web-based platforms such as Wikipedia, Twitter, YouTube, and FB, having confidence that the information is correct (Q16). Moreover, 15% (10/65) of patients and 26% (17/65) of relatives reported that wrong information received on the Web had negative effects on everyday life and the control of the disease (Q8). Brown et al [49] reported how doctors see and use social media. The findings of their study are comparable with our results showcasing insecurities for medical professionals and patients alike, regarding the reliability of information received on social media platforms.

Furthermore, we examined the influence of the ESA group on the patients’ selection of therapeutic regimens. We found that contents shared in the ESA group had relevant impact on the selection of treatment protocols, hospitals, and specialists. In this study, 20% of patients and 21% of relatives agreed a little or a lot that the group ESA affected their choice of treatment (Q5). Moreover, 19% of all patients reported that the group affected their selection of specialists (Q6). These statements indicate that FB has become an important source of information for patients with Ewing sarcoma and is affecting their treatment. These results underline the relevance of FB for patients with Ewing sarcoma and their treatment of choice [2,5,11,38].

At first sight, results of Q5-Q8 seem to be contradictory to the results of Q14-Q18 where there is a higher mean level of agreement to the statements made. However, the correlational analysis showed a significant positive correlation of all results (*P*<.001). For instance, the information received from FB did not affect the choice of treatment. The reason for this might be that the attending physicians mainly influence the choice of treatment. However, the Web could still be an important primary tool to look for medical information and users could trust the given information about medical conditions like Ewing sarcoma on social media platforms.

The survey results comparing patients and relatives were similar. The only significant difference was found in question 4 (Q4: I recommend the Facebook group “Ewing sarcoma awareness” in other social networks or to other affected people). Patients achieved significantly (*P*=.003) higher results. It can be estimated that patients who are going through the whole course of the disease feel more motivated to include others who are affected. This is in line with statements by Cutrona et al [50] who observed that many adults are willing to use e-communication or email to promote and report cancer screening to peers.

Our post on the ESA group’s home page, where we invited all group members to participate in our Web-based survey, resulted in controversial reactions and started a lively debate among ESA group members. After reacting to critical posts and clarifying the survey’s credibility and intention of our research work, the number of participants increased.

### Limitations

A limitation of our study is the small sample size of the study group. Because of FB’s regulations we were unable to send all group members a request (via private message) to answer our survey. Facebook does not allow mass messages. Messages to people you are not connected with usually end up in the FB spam folder. The only way of attracting attention for our questionnaire was by posting on the group’s home page. Unfortunately, only members who are frequently following the group’s activities were able to see our contribution. A much longer study period might have increased the number of participants. Because of a lack of previous studies on the topic, a sample size calculation was not possible.

A total of 27% of all participants of our survey stated that they visit the ESA group monthly or less and, a total of 183 different ESA group members posted contributions on the group’s home page. According to these numbers it can be estimated that only a relatively small number of users compared with the total number of group members (n=2227) is actively involved in the processes of the group. Another drawback is that information flow (via private message) between ESA group members was not visible to us and could therefore not be analyzed.

Because a larger number of group members were US citizens, it can be expected that the outcomes of other geographical populations differ from our results.

### Practical Implications

Our findings suggest that FB is an important platform for many patients with Ewing sarcoma and their relatives. Because of the disease’s low incidence, the most comfortable and simplest way to get in touch with other patients might be via FB. Peer-to-peer communication seems to enable considerable support for patients and their relatives.

We believe that implementing interaction tools on FB can benefit patients and their social environment and help individuals deal with the diagnosis of Ewing sarcoma. Web-based communication on FB with others who are affected can be implemented in the multidisciplinary therapeutic regimen for patients with Ewing sarcoma.

However, incorrect medical information received on the Web is an evident weakness that FB groups have. Therefore, we suggest cautious application of health-related information found in FB groups.

### Conclusions

In summary, the FB group ESA has a relevant impact on group members regarding treatment selection and in getting support through everyday life. Although the reliability and quality of information obtained from the Web is considered diverse, we believe that online forums are feasible tools for patients and relatives that help individuals not only find support and backing but also to share their experiences. The impact of Facebook regarding patients with Ewing sarcoma and their relatives who join such groups on the Web might be underestimated in traditional medical treatment regimens.

Reflecting on our results, we believe that questionnaires on social media platforms such as Facebook are suitable for a variety of scientific research questions in the future. Statements made in the group are in part questionable from a medical point of view and its impact on patient’s care needs further evaluation.

## Abbreviations

ESA: Ewing Sarcoma Awareness
FB: Facebook
Q: question
SD: standard deviation

## References

[1] Fox S (2008). Pew Research Center.

[2] Moorhead SA, Hazlett DE, Harrison L, Carroll JK, Irwin A, Hoving C (2013). A new dimension of health care: systematic review of the uses, benefits, and limitations of social media for health communication. J Med Internet Res.

[3] Hoffman-Goetz L, Donelle L, Thomson MD (2009). Clinical guidelines about diabetes and the accuracy of peer information in an unmoderated online health forum for retired persons. Inform Health Soc Care.

[4] Kreps GL, Neuhauser L (2010). New directions in eHealth communication: opportunities and challenges. Patient Educ Couns.

[5] Vance K, Howe W, Dellavalle RP (2009). Social internet sites as a source of public health information. Dermatol Clin.

[6] Sudau F, Friede T, Grabowski J, Koschack J, Makedonski P, Himmel W (2014). Sources of information and behavioral patterns in online health forums: observational study. J Med Internet Res.

[7] Lau AS (2011). Hospital-based nurses' perceptions of the adoption of Web 2.0 tools for knowledge sharing, learning, social interaction and the production of collective intelligence. J Med Internet Res.

[8] Gholami-Kordkheili F, Wild V, Strech D (2013). The impact of social media on medical professionalism: a systematic qualitative review of challenges and opportunities. J Med Internet Res.

[9] Farmer AD, Bruckner HC, Cook M, Hearing S (2009). Social networking sites: a novel portal for communication. Postgrad Med J.

[10] Greenhalgh T (2009). Patientpublic involvement in chronic illness: beyond the expert patient. BMJ.

[11] McMullan M (2006). Patients using the Internet to obtain health information: how this affects the patient-health professional relationship. Patient Educ Couns.

[12] (2015). Statista.

[13] (2010). Alexa.

[14] (2015). Facebook.

[15] Jha A, Lin L, Savoia E (2016). The Use of Social Media by State Health Departments in the US: Analyzing Health Communication Through Facebook. J Community Health.

[16] Thompson LA, Dawson K, Ferdig R, Black EW, Boyer J, Coutts J, Black NP (2008). The intersection of online social networking with medical professionalism. J Gen Intern Med.

[17] Fox S, Jones S (2009). Pew Research Center.

[18] Bender JL, Jimenez-Marroquin M, Jadad AR (2011). Seeking support on facebook: a content analysis of breast cancer groups. J Med Internet Res.

[19] Schumacher KR, Stringer KA, Donohue JE, Yu S, Shaver A, Caruthers RL, Zikmund-Fisher BJ, Fifer C, Goldberg C, Russell MW (2014). Social media methods for studying rare diseases. Pediatrics.

[20] Abramson K, Keefe B, Chou WS (2015). Communicating about cancer through Facebook: a qualitative analysis of a breast cancer awareness page. J Health Commun.

[21] Gaspar N, Hawkins DS, Dirksen U, Lewis IJ, Ferrari S, Le DM, Kovar H, Grimer R, Whelan J, Claude L, Delattre O, Paulussen M, Picci P, Sundby HK, Ladenstein R, Michon J, Hjorth L, Judson I, Luksch R, Bernstein ML, Marec-Bérard P, Brennan B, Craft AW, Womer RB, Juergens H, Oberlin O, van den Berg Hendrik (2015). Ewing Sarcoma: Current Management and Future Approaches Through Collaboration. J Clin Oncol.

[22] Cote GM, Choy E (2013). Update in treatment and targets in Ewing sarcoma. Hematol Oncol Clin North Am.

[23] Seker MM, Kos T, Ozdemir N, Seker A, Aksoy S, Uncu D, Zengin N (2014). Treatment and outcomes of Ewing sarcoma in Turkish adults: a single centre experience. Asian Pac J Cancer Prev.

[24] Lenhart A (2009). Pew Research Center.

[25] Duggan M, Brenner J Pew Research Center.

[26] Ramo DE, Thrul J, Chavez K, Delucchi KL, Prochaska JJ (2015). Feasibility and Quit Rates of the Tobacco Status Project: A Facebook Smoking Cessation Intervention for Young Adults. J Med Internet Res.

[27] Leithner A, Maurer-Ertl W, Glehr M, Friesenbichler J, Leithner K, Windhager R (2010). Wikipedia and osteosarcoma: a trustworthy patients' information?. J Am Med Inform Assoc.

[28] Burcher N (2008). Nickburcher.

[29] (2014). SurveyMonkey.

[30] Eysenbach G (2004). Improving the quality of Web surveys : the Checklist for Reporting Results of Internet E-Surveys (CHERRIES). J Med Internet Res.

[31] Greene JA, Choudhry NK, Kilabuk E, Shrank WH (2011). Online social networking by patients with diabetes: a qualitative evaluation of communication with Facebook. J Gen Intern Med.

[32] Gajaria A, Yeung E, Goodale T, Charach A (2011). Beliefs about attention-deficit/hyperactivity disorder and response to stereotypes: youth postings in Facebook groups. J Adolesc Health.

[33] Farmer AD, Bruckner H, Cook MJ, Hearing SD (2009). Social networking sites: a novel portal for communication. Postgrad Med J.

[34] De la Torre-Díez I, Díaz-Pernas FJ, Antón-Rodríguez M (2012). A content analysis of chronic diseases social groups on Facebook and Twitter. Telemed J E Health.

[35] Hale TM, Pathipati AS, Zan S, Jethwani K (2014). Representation of health conditions on Facebook: content analysis and evaluation of user engagement. J Med Internet Res.

[36] Lampe C, Vitak J, Gray R, Ellison N (2012). Perceptions of Facebook's value as an information source.

[37] Hale TM, Pathipati AS, Zan S, Jethwani K (2014). Representation of health conditions on Facebook: content analysis and evaluation of user engagement. J Med Internet Res.

[38] Cline R, Haynes K (2001). Consumer health information seeking on the Internet: the state of the art. Health Educ Res Dec.

[39] Pennbridge J, Moya R, Rodrigues L (1999). Questionnaire survey of California consumers' use and rating of sources of health care information including the Internet. West J Med.

[40] Cobb NK, Graham AL, Abrams DB (2010). Social network structure of a large online community for smoking cessation. Am J Public Health.

[41] Chou WS, Hunt YM, Beckjord EB, Moser RP, Hesse BW (2009). Social media use in the United States: implications for health communication. J Med Internet Res.

[42] Kontos EZ, Emmons KM, Puleo E, Viswanath K (2010). Communication inequalities and public health implications of adult social networking site use in the United States. J Health Commun.

[43] Lenhart A (2009). Pew Research Center.

[44] Davison KP, Pennebaker J, Dickerson S (2000). Who talks? The social psychology of illness support groups. Am Psychol.

[45] Moreno M, Parks M, Richardson L (2007). What are adolescents showing the world about their health risk behaviors on MySpace? MedGenMed. MedGenMed.

[46] Baptist AP, Thompson M, Grossman KS, Mohammed L, Sy A, Sanders GM (2011). Social media, text messaging, and email-preferences of asthma patients between 12 and 40 years old. J Asthma.

[47] Schippinger M, Ruckenstuhl P, Friesenbichler J, Leithner A (2014). [Osteosarcoma: reliability and quality of the information in the internet]. Wien Med Wochenschr.

[48] Donaldson L (2003). Expert patients usher in a new era of opportunity for the NHS. BMJ.

[49] Brown J, Ryan C, Harris A (2014). How doctors view and use social media: a national survey. J Med Internet Res.

[50] Cutrona SL, Roblin DW, Wagner JL, Gaglio B, Williams AE, Torres SR, Field TS, Mazor KM (2013). Adult Willingness to Use Email and Social Media for Peer-to-Peer Cancer Screening Communication: Quantitative Interview Study. JMIR Res Protoc.

